# Supplementary material for: Eliciting and modeling emotional requirements: a systematic mapping review
Source: PeerJ Comput Sci. 2024 Jan 19;10:e1782. doi: 10.7717/peerj-cs.1782 (PMC10803089; doi:10.7717/peerj-cs.1782)
Supplement: Supplemental Information 1 [file peerj-cs-10-1782-s001.docx]

**Appendix**

| **Study ID** | **Title** | **Ref.** |
| --- | --- | --- |
| PS 01 | Feelings and physics: Emotional, psychological, and other soft human requirements, by model-based systems engineering | [49] |
| PS 02 | A study of emotions in requirements engineering | [27] |
| PS 03 | Using the affect grid to measure emotions in software requirements engineering | [28] |
| PS 04 | Emotional requirements engineering | [5] |
| PS 05 | Requirement elicitation in product emotional design using fuzzy clustering and fuzzy cognitive model | [29] |
| PS 06 | Emotion-led modelling for people-oriented requirements engineering: The case study of emergency systems | [15] |
| PS 07 | Latent customer needs elicitation by use case analogical reasoning from sentiment analysis of online product reviews | [45] |
| PS 08 | Improving IS development teams' performance during requirement analysis in project—The perspectives from shared mental model and emotional intelligence | [17] |
| PS 09 | Participatory design-based requirements elicitation involving people living with dementia towards a home-based platform to monitor emotional well-being | [35] |
| PS 10 | Requirement engineering meets emotion: A case study of quiz MASTer | [51] |
| PS 11 | A method for eliciting and representing emotional requirements: Two case studies in e-healthcare | [31] |
| PS 12 | Don't worry, be happy - Exploring users' emotions during app usage for requirements engineering | [32] |
| PS 13 | Using machine learning to convey emotions during requirements elicitation interviews | [25] |
| PS 14 | Towards a requirements language for modeling emotion in videogames | [50] |
| PS 15 | Design of a Remote Emotional Requirement Elicitation Feedback Method | [42] |
| PS 16 | Aspect-sentiment-guided opinion summarization for user need elicitation from online reviews | [52] |
| PS 17 | Mining Customer Requirement from Online Reviews Based on Multi-aspected Sentiment Analysis and KANO Model | [26] |
| PS 18 | Integrating SSTQUAL, Kano Model and Attractiveness Engineering to Analyze User’s Emotional Needs in Self Check-in Service | [36] |
| PS 19 | Emotion-oriented requirements engineering: A case study in developing a smart home system for the elderly | [12] |
| PS 20 | Emotional attachment framework for people-oriented software | [22] |
| PS 21 | Value-based requirements engineering: method and experience | [16] |
| PS 22 | Theory of Constructed Emotion Meets RE | [43] |
| PS 23 | Using Work System Design, User Stories and Emotional Goal Modeling for an mHealth System | [44] |
| PS 24 | Emotional Requirements for Well-being Applications : The Customer Journey | [2] |
| PS 25 | On the Road to Enriching the App Improvement Process with Emotions | [30] |
| PS 26 | Improving the Identification of Hedonic Quality in User Requirements ‚A Controlled Experiment | [20] |
| PS 27 | User-oriented requirements engineering | [23] |
| PS 28 | Visualizing Emotional Requirements | [38] |
| PS 29 | Requirements in Conflict: Player vs. Designer vs. Cheater | [39] |
| PS 30 | Branding and Communication Goals for Content-Intensive Interactive Applications | [24] |
| PS 31 | Emotional Requirements in Video Games | [37] |
| PS 32 | Investigating the Role of 'Soft Issues' in the RE Process | [46] |
| PS 33 | Use of personal values in requirements engineering–a research preview | [13] |
| PS 34 | Investigating the influence of personal values on requirements for health care information systems | [14] |
| PS 35 | Viewpoint modelling with emotions: a case study | [48] |
| PS 36 | Balancing security requirements and emotional requirements in video games | [40] |
| PS 37 | Augmenting emotional requirements with emotion markers and emotion prototypes | [19] |
| PS 38 | Emotional requirements | [41] |
| PS 39 | Supporting worth mapping with sentence completion | [18] |
| PS 40 | Requirements Engineering for Organizational Transformation | [21] |
| PS41 | Incorporating Multimodal Sentiments into Conversational Bots for Service Requirement Elicitation | [34] |
| PS42 | Toward Emotion-Oriented Requirements Engineering: A Case Study of a Virtual Clinics Application | [33] |
| PS43 | Teaching motivational models in agile requirements engineering. | [53] |
| PS44 | Multi-Modal Emotion Recognition for Enhanced Requirements Engineering: A Novel Approach | [54] |
| PS45 | Engineering Emotional Requirements for Interactive Digital Narratives | [55] |
| PS46 | Theory of constructed emotion meets RE: An industrial case study. | [56] |
